# Care after cataract surgery in Nairobi, Kenya

**Published:** 2016

**Authors:** George S Odhiambo Ohito

**Affiliations:** Is a cataract surgeon at St Mary's Mission Hospital in Langata, Nairobi.

**Figure:**
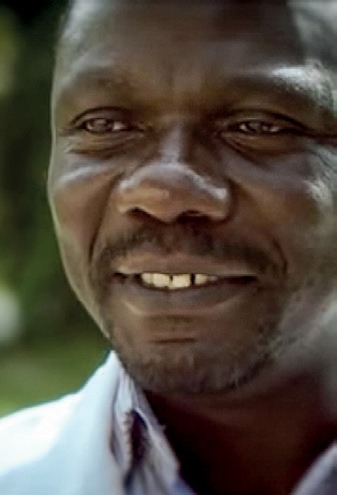
George S Odhiambo Ohito

Postoperative care of the cataract patient is an integral part of cataract management with the objectives of minimising patient discomfort and pain, preventing injury and complications, and improving surgical and vision outcomes. It need not be so restrictive as to cause patients to have a significant lifestyle change.

Postoperative care is planned and discussed with the patient before surgery, and advice is adapted to fit the circumstances of each patient. For example, to insist that an old widow who lives alone in a rural area does not cook her food using a firewood stove, is to deny her meals for the recovery period. Her only option would be to ‘break the rules’, which might in turn reduce her confidence in the advice and treatment regime she has been given, for example regarding the use of medication.

At St Mary's Mission Hospital in Nairobi, almost all operations are done as day cases and we have limited the postoperative visits to three, unless complications arise. Visits take place on day 1 (the first day after surgery), day 8 (or 1 week) and at 6-8 weeks (for a final examination and refraction). We have an autorefractor and aim to refract all patients at 6–8 weeks. In the event of any complications, the visits may be increased as necessary.

Immediately after surgery, we advise adult patients to take 1,000 mg of paracetamol, repeated every 8 hours for 1–2 days, as needed. The dressing is left on until the day after surgery and is only removed by the clinic staff. On the first day after surgery (day 1), we remove the dressing, clean the eye and check the eye for complications. If there are none, we prescribe a combined steroid/antibiotic drop to reduce inflammation and as a prophylaxis against bacterial infection. (We avoid ointments as they can temporarily affect the vision.) We do not routinely prescribe cycloplegics unless there is significant fibrinoid reaction. After the first week, patients are given plain steroid eyedrops to use at home for 2–3 weeks.

We have simplified the postoperative instructions to patients as follows:

- No special diet, eat your normal meals.
- Have a bath, just avoid splashing water directly into the eye, and avoid soap on the face for two weeks.
- Watch TV if comfortable.
- Use dark glasses (sunglasses) if you can afford a pair.
- Avoid strenuous exercise or heavy work for at least a month.

Generally, we encourage an early return to normal life.

